# Optimisation of the foot-and-mouth disease virus 2A co-expression system for biomedical applications

**DOI:** 10.1186/1472-6750-13-67

**Published:** 2013-08-22

**Authors:** Ekaterina Minskaia, John Nicholson, Martin D Ryan

**Affiliations:** 1 Biomedical Sciences Research Complex, North Haugh, University of St.Andrews, St.Andrews, KY16 9ST Fife, Scotland, UK

**Keywords:** Protein co-expression, Bicistronic vector, Co-translational ‘self-cleavage’

## Abstract

**Background:**

Many biomedical applications require the expression or production of therapeutic hetero-multimeric proteins/protein complexes: in most cases only accomplished by co-ordinated co-expression within the same cell. Foot-and-mouth disease virus 2A (F2A) and ‘2A-like’ sequences are now widely used for this purpose. Since 2A mediates a co-translational ‘cleavage’ at its own C-terminus, sequences encoding multiple proteins (linked via 2As) can be concatenated into a single ORF: a single transgene. It has been shown that in some cases, however, the cleavage efficiency of shorter versions of F2A may be inhibited by the C-terminus of certain gene sequences immediately upstream of F2A. This paper describes further work to optimise F2A for co-expression strategies.

**Results:**

We have inserted F2A of various lengths in between GFP and CherryFP ‘reporter’ proteins (in reciprocal or tandem arrangements). The co-expression of these proteins and cleavage efficiencies of F2As of various lengths were studied by *in vitro* coupled transcription and translation in rabbit reticulocyte lysates, western blotting of HeLa cell lysates and fluorescence microscopy.

**Conclusions:**

Optimal and suboptimal lengths of F2A sequences were identified as a result of detailed ‘fine-tuning’ of the F2A sequence. Based on our data and the model according to which 2A activity is a product of its interaction with the exit tunnel of the ribosome, we suggest the length of the F2A sequence which is not ‘sensitive’ to the C-terminus of the upstream protein that can be successfully used for co-expression of two proteins for biomedical applications.

## Background

Many biomedical applications rely on the ability to co-express multiple proteins within the same cell. These may include co-expression of genes encoding subunits of hetero-multimeric therapeutic proteins (e.g. immunoglobulins, cell receptors, interleukins, enzymes, transcription factors), multiple therapeutic genes for a combined and/or synergistic effects (e.g. immune-stimulatory), and co-expression of therapeutic genes plus required co-factor or marker gene (e.g. drug resistance, colour, fluorescence) for detection or selection of transduced cells (reviewed by de Felipe [1] and Pfutzner [2]).

Various conventional approaches for protein co-expression have been utilized such as the use of multiple internal promoters, internal ribosome entry sites (IRESes), messenger RNA splicing, fusion proteins, and post-translational proteolysis. There are, however, disadvantages associated with each of these approaches. For example, steric effects often lead to loss of function of fused proteins. In order to avoid such problems, proteinase cleavage sites can be incorporated. In this case both the polyprotein substrate and the processing enzyme must, however, be co-expressed in the same subcellular site. When multiple promoters have been employed, interference has occurred between promoters leading to promoter suppression and rearrangement. IRES elements, identified both in viral and cellular eukaryotic mRNAs, differ in length (from 130 bp to 1 kb). The most efficient viral IRESes successfully utilized in vectors used for biomedical purposes are about 500 bp. However, their size can cause problems in packaging within size-restricted vectors commonly used for biomedical applications such as adeno-associated and retroviral vectors. Furthermore, expression from IRESes is dependent on various cellular binding factors which vary amongst different cell types. Critically, the protein downstream of the IRES is only expressed to ~10% of that upstream [1-7].

*Picornavirus* (foot-and-mouth disease virus; FMDV) F2A and ‘2A-like’ sequences are now widely used for co-expression of multiple genes. The 2A region of the FMDV encodes a sequence that mediates self-processing by a translational effect variously referred to as ‘ribosome skipping’, ‘stop-go’ and ‘stop-carry on’ translation [8,9]. Analysis of recombinant polyproteins and artificial polyprotein systems in which 2A was inserted between two reporter proteins showed that the FMDV 2A oligopeptide (plus the N-terminal proline of the 2B downstream protein) co-translationally ‘self-cleaved’ at the glycyl-prolyl pair site corresponding to the 2A/2B junction (−LLNFDLLKLAGDVESNPG^↓^P-). The use of longer versions of 2A with N-terminal extensions derived from FMDV capsid protein 1D upstream of 2A (~30aa in total) was reported to produce higher levels of cleavage [10-13].

For simplicity, the co-translational ribosome skipping event will be referred to as ‘cleavage’. The merits of this system are: (i) co-expression of proteins linked by 2A is independent of the cell type (since cleavage activity is only dependent on eukaryotic ribosomes, structurally highly conserved amongst the eukaryota), (ii) multiple proteins are co-expressed in equimolar amounts from a single transcript mRNA (single ORF) under the control of only one promoter and, (iii) 2A is smaller (54-174 bp) compared to IRES elements. This makes this unique sequence an attractive substitute for previously used approaches for co-expression of multiple genes [10,14-16]. However, it should be noted that (i) 2A remains as a C-terminal extension of the upstream product, and (ii) proline forms the N terminus of the downstream protein.

To date, various reporter proteins together with proteins requiring discrete co- and post-translational subcellular localization have been successfully co-expressed in *in vitro* and *in vivo* heterologous systems using F2A sequences of various lengths [11,17-24]. However, some limitations of using this strategy were reported. Firstly, whilst the longest F2A sequence (58aa) tested to date was shown to produce the most efficient cleavage, the C-terminal F2A extension of the upstream translation product may have an effect on protein confirmation and activity - such as in the production of monoclonal antibodies or expression of enzymes. To minimise this effect, a number of laboratories have used shorter versions of F2As [11,14,19,22,25], or, incorporated a furin cleavage site between the C-terminus of the upstream protein and N-terminus of the 2A sequence such that the C-terminal extension is ‘trimmed’ away. This approach can only be used for secreted proteins, however, since furin is primarily localised within the Golgi apparatus [22,26].

It has been reported, however, that the efficiency of F2A cleavage may be greatly inhibited in certain cases where the C-terminal sequences of the upstream gene were shown to lower F2A activity [27,28]. It was proposed that such inhibition could be overcome by the use of different lengths of the sequence between the C-terminal region and F2A [11]. To further investigate F2A cleavage efficiency and to optimise the 2A system for the coordinate expression of two genes from a bicistronic vector, we generated constructs encoding two fluorescent ‘reporter’ proteins, GFP and CherryFP, in different positions linked by F2A sequences of various lengths. The co-expression of these cytoplasmic proteins and the comparative cleavage efficiencies were determined both *in vivo* in HeLa cells, a human cell line most commonly used in biomedical applications (western blotting/fluorescence microscopy), and *in vitro* (coupled transcription/translation system).

## Results

### Co-expression of GFP and CherryFP fluorescent proteins from pGFP-F2A-CherryFP constructs

**Figure 1:**
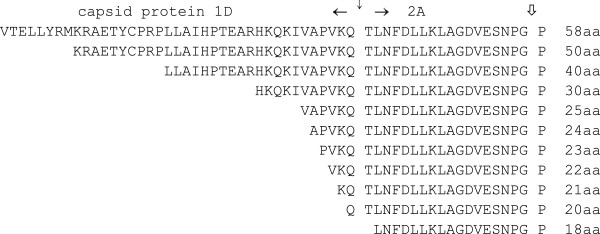
**F2A sequences used for co-expression of fluorescent ‘reporter’ proteins.** 2A mediates the co-translational ‘ribosome skipping’ event whilst fused to the upstream capsid protein 1D. Subsequently, 2A is co-translationally cleaved from 1D by an FMD virus proteinase (3C^pro^) which (artificially) defines the length of 2A: the functional entity is, however, longer than this 18aa tract. The 3C^pro^ cleavage is indicated by the solid vertical arrow, the site of the 2A-mediated ribosome skipping event is shown by the open vertical arrow. Versions of various lengths were used by producing truncations of the 58aa version.

**Figure 2:**
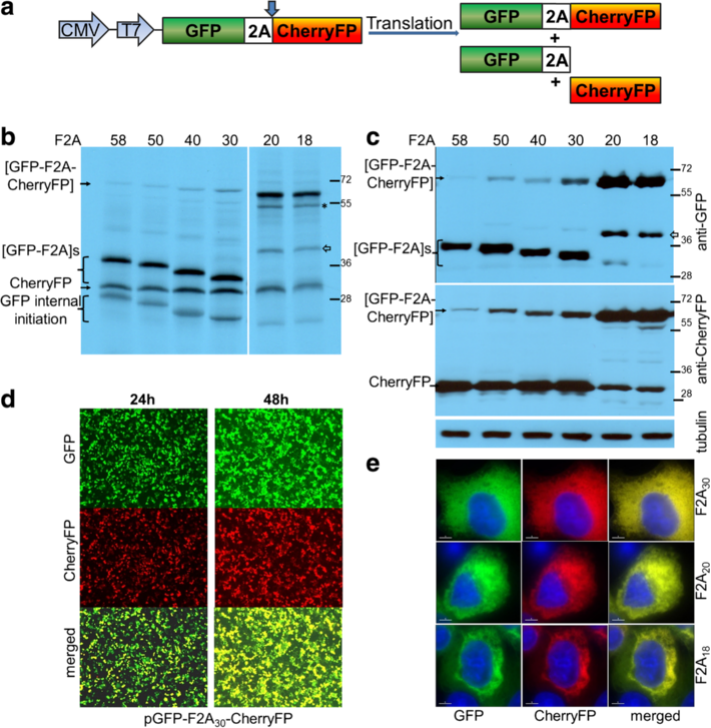
**Efficiency of F2A cleavage in GFP-F2A-CherryFP context.** F2A sequences of various lengths were used to co-express GFP and CherryFP proteins from pGFP-F2A-CherryFP constructs (**a**, schematic presentation) *in vitro* using *TnT* coupled transcription/ translation rabbit reticulocyte lysates **(b)** and transfected HeLa cells **(c)**. For *TnT*, reticulocyte lysates were programmed with 20 ng of plasmid DNA and translation products were resolved by the 12% SDS-PAGE. For *in vivo* studies, HeLa cells were transfected with 1.5 μg of plasmid DNA and harvested 30 h post transfection. Cells were lysed in RIPA buffer and equal amounts of total protein for each transfection were loaded onto 12% SDS-PAGE gel. The proteins were transferred onto a nitrocellulose membrane, blocked in PBST containing 5% milk and probed with anti-GFP (upper blot) and anti-CherryFP (lower blot) antibodies overnight at 4°C. Detection of bound primary antibody was achieved by using respective secondary antibodies, followed by ECL detection. **(d)** Expression of fluorescent proteins was detected in transfected HeLa cells at 24 h and 48 h post transfection in Evos microscope using 4 × objective. **(e)** Cellular co-localisation of [GFP-F2A] and CherryFP 30 h post-transfection. HeLa cells pre-plated on cover slips were transfected with 0.5 μg of plasmid DNA and incubated for 30 h, fixed with 100% methanol and mounted using VECTASHIELD mounting medium with DAPI. The images were acquired with Deltavision microscope using 100 × objective using Resolve 3D software. All experiments were done in triplicates.

A series of constructs were produced encoding GFP and CherryFP fluorescent ‘reporter’ proteins under the control of both CMV and T7 promoters. Sequences encoding GFP and CherryFP were separated by either the longest F2A sequence (F2A_58_) or N-terminally truncated versions of various lengths (F2A_50_, F2_40_, F2A_30_, F2A_20_, and F2A_18_; Figure 1), to establish the optimal length of F2A sequence for co-expression of two proteins and their relative cleavage efficiencies. The expected translation products were cleaved [GFP-F2A] and CherryFP, and the uncleaved polyprotein [GFP-F2A-CherryFP] (Figure 2a).

For the *in vitro* translation studies, rabbit reticulocyte lysates were programmed with the pGFP-F2A-CherryFP series of constructs and the translation products resolved by 12% SDS-PAGE (Figure 2b). As expected, two major translation products (of the expected molecular weights) were observed. In each track the upper slower migrating product corresponded to either [GFP-F2A_58_] (34.5 kDa), [GFP-F2A_50_] (33.5 kDa), [GFP-F2A_40_] (32.3 kDa) or [GFP-F2A_30_] (31.2 kDa). The second product corresponded to the CherryFP cleavage product. Translation profiles derived from pGFP-F2A-CherryFP constructs also showed a low level of internal initiation which produced products of lower molecular weights. The bands, based on the gradual decrease in molecular weight, were comparable to the [GFP-F2A] cleavage products and correspond to products produced by leaky scanning, initiating within GFP at Met88, in favourable Kozak context (cC**AUG**c, indicated as ‘GFP internal initiation’ in Figure 2b).

HeLa cells were transfected with this panel of constructs and cell lysates analysed 30 h post transfection. Similarly, the two major cleavage products were detected by western blotting either with an anti-GFP antibody showing [GFP-F2A]s of decreasing size (Figure 2c, upper blot), or, an anti-CherryFP antibody (Figure 2c, lower blot). Uncleaved polyproteins were also detected, their accumulation increasing as the length of F2A decreased, being highest for the pGFP-F2A_30_-CherryFP construct. These data, together with our *in vitro* analysis, demonstrated that the cleavage efficiency is slightly reduced when a shorter version (F2A_30_) is used, compared to that observed for the 58aa version.

Fluorescence imaging was used to directly visualise co-expression of fluorescent proteins. To demonstrate that GFP and CherryFP proteins linked via F2A were co-expressed within the same cell, we transfected HeLa cells with the panel of pGFP-F2A-CherryFP constructs (F2A_58_, F2A_50_, F2A_40_ and F2A_30_). In each case transfected cells efficiently co-expressed both GFP and CherryFP for up to 48 h post-transfection (Figure 2d; the images presented for pGFP-F2A_30_-CherryFP were representative of all constructs), with both proteins localised to the cytoplasm of transfected cells (Figure 2e). We could not detect any effect of increasing the length of the C-terminal extension of GFP by F2A (30-58aa) upon fluorescence.

In contrast to our constructs with F2A of 58–30 amino acids, translation directed by plasmids pGFP-F2A_20_-CherryFP and pGFP-F2A_18_-CherryFP showed three major translation products (Figure 2b). In each gel track, the most slowly migrating band corresponded to the uncleaved polyprotein forms [GFP-F2A_20_-CherryFP] and [GFP-F2A_18_-CherryFP] of the expected molecular weights (~57 kDa). The bands corresponding to the two cleavage products, [GFP-F2A_20_] or [GFP-F2A_18_] and CherryFP could not be resolved due to the similar molecular weights. In addition, three extra minor products were observed in each of these translation reactions. Internal initiation within the upstream GFP gene (probably Met88) produced both the fastest migrating cleaved product, corresponding to protein of ~20 kDa which was almost undetectable on the gel (indicated in Figure 2b as ‘GFP internal initiation’) and the second slow migrating uncleaved product of ~50 kDa (highlighted with asterisk). The third slower migrating minor product corresponded to a protein of unknown identity (shown with white arrow), although we suspect that this represents a translation product with a C-terminal adduct of tRNA.

Our *in vivo* studies were consistent with the *in vitro* data: the western blotting data demonstrating that the major products were the uncleaved [GFP-F2A_20_-CherryFP] and [GFP-F2A_18_-CherryFP] polyproteins (Figure 2c). Small amounts of the CherryFP cleavage product, compared to F2A_58_-_30_-based constructs, were still detectable with anti-CherryFP antibody (Figure 2c, lower blot), the upstream cleavage products were, however, either almost undetectable [GFP-F2A_20_] or absent [GFP-F2A_18_] (Figure 2c, upper blot). It is important to note that the amounts of proteins observed in these blots is a function of detection using two quite different antibodies, raised against either GFP or CherryFP and they have different affinities for their antigens. Interestingly, extracts of HeLa cells transfected with plasmids pGFP-F2A_20_-CherryFP and pGFP-F2A_18_-CherryFP also contained an unexpected protein with an estimated size of ~45 kDa (shown with white arrow), similar to that detected in reticulocyte lysates, which reacted with the anti-GFP, but not anti-CherryFP antibodies. The nature of this protein, which could be generated by a leaky scanning mechanism, or, be a product of degradation of the uncleaved precursor is under further investigation.

Interestingly, in the case of constructs which produced a high level of cleavage, the fluorescent signals from both GFP and CherryFP were distributed throughout the cytoplasm, in the case of constructs which produced low levels of cleavage, the fluorescent signals were accumulated mostly around the nucleus (Figure 2e).

### Tandem repeated cherry fluorescent proteins

**Figure 3:**
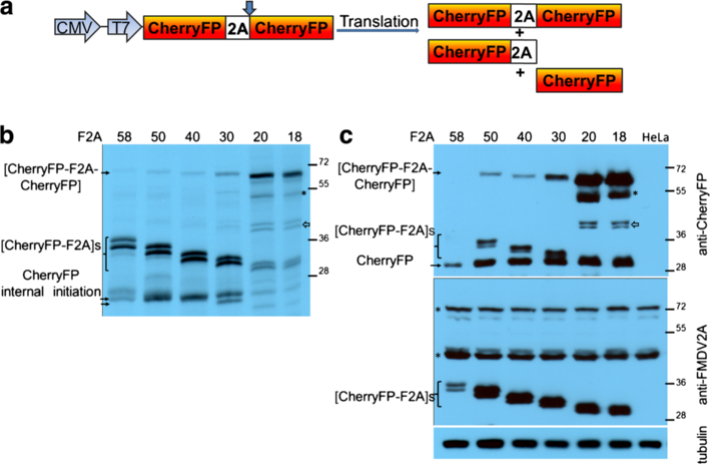
**Efficiency of F2A cleavage in CherryFP-F2A-CherryFP context.** F2A sequences of various lengths were used to co-express two CherryFP proteins from pCherryFP-F2A-CherryFP constructs (**a**, schematic presentation) *in vitro* using coupled transcription/ translation rabbit reticulocyte lysates **(b)** and transfected HeLa cells **(c)**. For *TnT*, reticulocyte lysates were programmed with 20 ng of plasmid DNA and translation products were resolved by the 12% SDS-PAGE. For *in vivo* studies, HeLa cells were transfected with 1.5 μg of plasmid DNA and harvested 30 h post transfection. Cells were lysed in RIPA buffer and equal amounts of total protein for each transfection were loaded onto 12% SDS-PAGE gel. The proteins were transferred onto a nitrocellulose membrane, blocked in PBST containing 5% milk and probed with anti-CherryFP (upper blot) and anti-FMDV2A (lower blot) antibodies overnight at 4°C. Detection of bound primary antibody was achieved by using respective secondary antibodies, followed by ECL detection. All experiments were done in triplicates.

To determine if the C-terminal region of the protein upstream of 2A affected the cleavage activity, we substituted the upstream gene (GFP) with that of CherryFP (Figure 3a). Again, these sequences were linked by either F2A_58_, F2A_50_, F2A_40_, F2A_30_, F2A_20_ or F2A_18_. Similar to our previous results, when reticulocyte lysates were programmed with constructs encoding F2As of diminishing lengths, the uncleaved polyprotein [CherryFP-F2A-CherryFP] formed a very minor translation product for F2A of 58 to 30aa (Figure 3b). In the case of these F2As, a doublet was observed corresponding to the [CherryFP-F2A] cleavage product. The fastest migrating product corresponded to the cleavage product downstream of F2A, CherryFP. The translation profiles derived from all pCherryFP-F2A-CherryFP constructs showed a high level of internal initiation, and produced minor translation products which corresponded in size to products from internal initiation at one of three start codons within the N-terminal CherryFP sequence (Met10, Met17 and Met23, with Met10 and Met23 being in more favourable Kozak contexts (AaC**AUG**G, ttC**AUG**c and caC**AUG**G, respectively; upper-case letters indicate highly conserved bases, and lower-case letters – bases that can vary). It should be noted that internal initiation at Met10 produces the same decrease in molecular mass as that observed when the length of 2A was designed in 10aa increments.

These observations *in vitro* were confirmed by our *in vivo* experiments. Cells were transfected with the panel of pCherryFP-F2A-CherryFP constructs and cleavage activities determined by western blotting (Figure 3c). As with the pGFP-F2A-CherryFP constructs, uncleaved [CherryFP-F2A_58, 50, 40_-CherryFP] polyproteins were barely detectable by the anti-CherryFP antibodies. The major products in these cases were the [CherryFP-F2A] and CherryFP cleavage products. Again, [CherryFP-F2A] migrated as a doublet, with the fastest migrating product corresponding to the CherryFP cleavage product - of uniform size across the gel tracks (Figure 3c, upper blot). Although easily detectable for the 30aa version, the uncleaved form accumulated to a somewhat lesser degree than the two major cleavage products. As we observed for the *in vitro* translations, the upstream cleavage products (now [CherryFP-F2A]) were detected by an anti-FMDV2A antibody (Figure 3c, lower blot), but the uncleaved [CherryFP-F2A-CherryFP] was not. Using this antibody, two non-specific proteins were detected both in transfected and mock-transfected cells (Figure 3c, lower blot, highlighted with asterisks). Interestingly, the amounts of the upstream and downstream cleavage products detected by both anti-CherryFP and anti-FMDV2A antibodies in HeLa cells transfected with pCherryFP-F2A_58_-CherryFP construct were lower than those expressed from constructs with shorter 2As.

Similar to our data obtained for pGFP-F2A_20_-CherryFP and pGFP-F2A_18_-CherryFP, both in reticulocyte lysates (Figure 3b) and in transfected HeLa cells (Figure 3c), the uncleaved polyproteins [CherryFP-F2A_20_-CherryFP] and [CherryFP-F2A_18_-CherryFP] represented the major translation products. The cleavage products upstream of F2A ([CherryFP-F2A_20_] and [CherryFP-F2A_18_]) migrated as doublets, as discussed above. The cleavage product downstream of F2A, CherryFP, was of uniform size across all the gel tracks. Interestingly, it appeared that cleavage was more efficient when CherryFP was upstream of 2A, rather than GFP – shown using both anti-CherryFP (Figure 3c, upper blot) and anti-FMDV2A antibodies (Figure 3c, lower blot). We assume the higher amounts of cleaved products arose from the changes in the C-terminal sequences of the upstream gene (CherryFP *versus* GFP), as the linker sequences connecting them to F2A were identical in all constructs (−RAKRSLE-). In addition, similar to the data presented for pGFP-F2A-CherryFP constructs, two extra bands were observed in both reticulocyte lysates and HeLa cells transfected with constructs containing F2As of 20 and 18 amino acids. The slow migrating band corresponded to protein produced by internal initiation within the upstream Cherry gene (probably Met71; Figure 3b, highlighted with asterisk). This product was also detected in HeLa cells using anti-CherryFP antibodies (Figure 3c, upper blot, highlighted with asterisk). The extra doublet (~45 kDa) we observed using reticulocyte lysates (Figure 3b, shown with white arrow) was also detected in HeLa cell extracts using anti-CherryFP antibodies (Figure 3c, upper blot, shown with white arrow), but not anti-FMDV 2A antibodies.

### Co-expression of CherryFP and GFP fluorescent proteins from pCherryFP-F2A-GFP constructs

**Figure 4:**
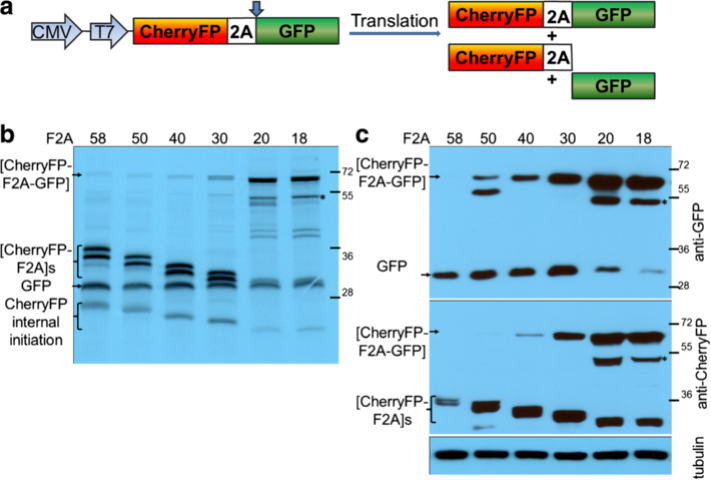
**Efficiency of F2A cleavage in CherryFP-F2A-GFP context.** F2A sequences of various lengths were used to co-express CherryFP and GFP proteins from pCherryFP-F2A-GFP constructs (**a**, schematic presentation) *in vitro* using coupled transcription/ translation rabbit reticulocyte lysates **(b)** and transfected HeLa cells **(c)**. For *TnT*, reticulocyte lysates were programmed with 20 ng of plasmid DNA and translation products were resolved by the 12% SDS-PAGE. For *in vivo* studies, HeLa cells were transfected with 1.5 μg of plasmid DNA and harvested 30 h post transfection. Cells were lysed in RIPA buffer and equal amounts of total protein for each transfection were loaded onto 12% SDS-PAGE gel. The proteins were transferred onto a nitrocellulose membrane, blocked in PBST containing 5% milk and probed with anti-GFP (upper blot) and anti-CherryFP (lower blot) antibodies overnight at 4°C. Detection of bound primary antibody was achieved by using respective secondary antibodies, followed by ECL detection. All experiments were done in triplicates.

The downstream CherryFP sequences were replaced with GFP to create pCherryFP-F2A-GFP, again linked with the different lengths of F2A (Figure 4a). The pattern of accumulation of uncleaved polyproteins was as observed before: an increase as the length of F2A decreases. The uncleaved polyprotein formed the major product in the case of pCherryFP-F2A_20_-GFP and pCherryFP-F2A_18_-GFP both in reticulocyte lysates (Figure 4b) and in HeLa cells (Figure 4c). The cleaved upstream proteins, [CherryFP-F2A]s, expressed from all six constructs, migrated as a doublet in the case of translation *in vitro* (Figure 4b) and *in vivo* - in the latter case detected by western blotting using anti-CherryFP antibodies (Figure 4c, lower blot). The second major band corresponded to the downstream cleavage product, GFP (Figure 4b and 4c, upper blot). Translation profiles derived from pCherryFP-F2A-GFP constructs with F2As_58-30_ showed a certain level of internal initiation (probably from Met71) within the upstream CherryFP gene. This produced fast migrating bands corresponding to cleaved products of decreasing molecular weights (Figure 4b, indicated as ‘CherryFP internal initiation’).

The F2A_20_ and F2A_18_ versions showed poor cleavage: the GFP cleavage product was almost undetectable in western blots using the anti-GFP antibody (Figure 4c, upper blot). Internal initiation was observed in the reticulocyte lysate translation *in vitro*. The faster migrating bands corresponded to N-terminally truncated [CherryFP-F2A] proteins (probably produced by initiation at Met71; Figure 4b, indicated as ‘CherryFP internal initiation’). Initiation at this site also accounts for products migrating somewhat more rapidly than the full-length (uncleaved) polyprotein (Figure 4, b and c, highlighted with asterisks). This extra product was observed in both reticulocyte lysates programmed with the plasmids encoding F2As of 20 and 18aa, and in extracts of HeLa cell transfected with these constructs. In these cases, the poor cleavage activity leads to an accumulation of uncleaved products and enabled sufficient material to accumulate to be observed directly (translation *in vitro*), or, be recognised by both anti-CherryFP and anti-GFP antibodies.

### ‘Fine-tuning’ of shorter F2A sequences

**Figure 5:**
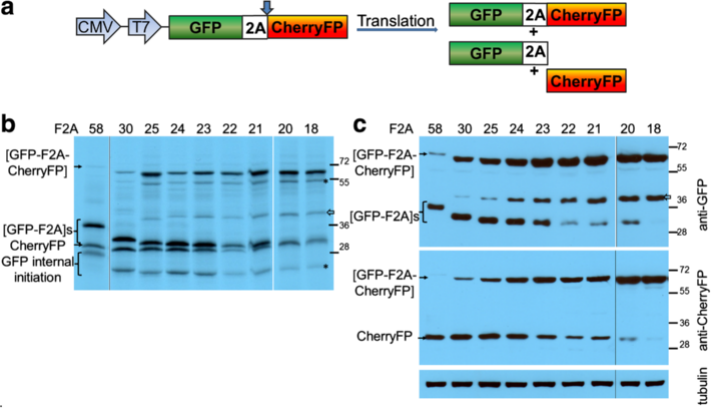
**‘Fine-tuning’ of F2A cleavage in GFP-F2A-CherryFP context.** F2A sequences of various lengths were used to co-express GFP and CherryFP proteins from pGFP-F2A-CherryFP constructs (**a**, schematic presentation) *in vitro* using coupled transcription/ translation rabbit reticulocyte lysates **(b)** and transfected HeLa cells **(c)**. For *TnT*, reticulocyte lysates were programmed with 20 ng of plasmid DNA and translation products were resolved by the 12% SDS-PAGE. For *in vivo* studies, HeLa cells were transfected with 1.5 μg of plasmid DNA and harvested 30 h post transfection. Cells were lysed in RIPA buffer and equal amounts of total protein for each transfection were loaded onto 12% SDS-PAGE gel. The proteins were transferred onto a nitrocellulose membrane, blocked in PBST containing 5% milk and probed with anti-GFP (upper blot) and anti-CherryFP (lower blot) antibodies overnight at 4°C. Detection of bound primary antibody was achieved by using respective secondary antibodies, followed by ECL detection. All experiments were done in triplicates.

To assess the utility of shorter F2A sequences for co-expression, we produced a series of constructs in which sequences encoding GFP and CherryFP ‘reporter’ proteins were linked by the panel of N-terminally single-residue truncated versions of F2A (F2As_25-21_; Figure 1). These constructs were analysed together with the previously described pGFP-F2A-CherryFP constructs containing F2A_58_, F2A_30_, F2A_20_ and F2A_18_. Based upon the data shown in Figure 2, pGFP-F2A_58_-CherryFP / pGFP-F2A_30_-CherryFP were used as exemplars of highly efficient 2A cleavage and pGFP-F2A_20_-CherryFP / pGFP-F2A_18_-CherryFP as exemplars of inefficient cleavage. Analysis of the translation products showed three major bands which corresponded to the three predicted translation products and their molecular weights: uncleaved [GFP-F2A-CherryFP] polyprotein and the two major cleavage products, [GFP-F2A] and CherryFP (Figure 5b). While the intensities of the latter bands were much higher in reticulocyte lysates programmed with constructs containing F2As of 30, 25, 24 and 23aa (in comparison with F2As of 22-18aa), the translation products derived from the four constructs encoding shorter 2A sequences were mostly uncleaved. Translation profiles derived from all constructs showed a low level of internal initiation (probably from Met88) within the upstream GFP gene. The fast migrating band corresponded to N-terminally truncated cleaved products [GFP-F2A] and slow migrating band corresponded to N-terminally truncated uncleaved polyprotein (Figure 5b, highlighted with asterisk). Again, the additional translation product of ~45KDa was observed (more prominent with F2A versions of 25aa and shorter), but was absent in constructs with F2A_30_ and F2A_58_. This additional translation product was detected by anti-GFP (Figure 5c, upper blot, shown with white arrow), but not anti-CherryFP antibodies (Figure 5c, lower blot). From F2A_25_, the intensity of this band increased as the length of F2A decreased. In the cases of F2A of 23aa and longer, the translation products upstream of F2A ([GFP-F2A]) and downstream ([CherryFP]) were easily detected by anti-GFP (Figure 5c, upper blot) and anti-CherryFP antibodies (Figure 5c, lower blot), respectively. In the case of F2As_22-20_, cleavage products were barely detectable, whilst for F2A_18_ no cleavage products could be detected.

## Discussion

Initial characterization of the FMDV 2A region by Ryan *et al*. [10] showed that while the FMDV 2A/2B cleavage activity was mediated by residues located within the 19 amino acid version of F2A, N-terminal truncation of F2A reduced cleavage efficiency. Ryan and Drew [14] further described the construction of plasmid (pCAT2AGUS) in which a 19 amino acid version of F2A was inserted between the reporter genes chloramphenicol acetyl transferase (CAT) and β-glucuronidase (GUS). Analyses of *in vitro* translation products derived from a series of constructs in which sequences were progressively deleted from the N-terminal region of F2A showed that activity was ablated in versions with less than 13 residues (−LKLAGDVESNPGP-). It was concluded that residues comprising the N-terminal portion of the F2A region ‘whilst being influential were not critical for cleavage activity’ [14].

According to the model for 2A-mediated cleavage, residues that may influence the activity are predicted to reside within the exit tunnel of the ribosome. The use of longer versions of F2A with N-terminal extensions derived from capsid protein 1D (to a total of ~30aa) were reported to produce higher levels of cleavage [10-13]. Specifically, in a series of constructs based upon pGFP2AGUS, N-terminal extension of 2A by 5aa of 1D (equivalent to F2A_25_) improved cleavage, but extension by 14, 21 and 39aa (equivalent of F2A of 35, 40 and 58aa) produced complete cleavage and an equal stoichiometry of both translation products *in vitro *[11].

These observations are consistent with our *in vitro* and *in vivo* data on cleavage efficiency in constructs expressing GFP and CherryFP fluorescent ‘reporter’ proteins separated by F2As of 58, 50, 40 and 30aa. For all constructs tested, the i*n vitro* analyses showed the polyprotein was efficiently cleaved, the upstream protein bearing the F2A ‘tail’ at its C-terminus. In transfected HeLa cells, however, small amounts of uncleaved polyprotein were detected by western blotting (using anti-GFP / anti-CherryFP, but not anti-FMDV2A, antibodies) in constructs encoding the F2A_30_ version. Swapping the gene order had no effect on cleavage efficiency when longer versions of 2As were used, showing that for these particular lengths, cleavage is not sensitive to the C- terminal sequence of the upstream protein - since this has already transited through the exit tunnel of the ribosome.

Further experiments demonstrated that the cleavage efficiency was gradually reduced with each N-terminal amino acid deletion, shown by both our *in vitro* and *in vivo* data when pGFP-F2A-CherryFP constructs with F2As of 25aa to 18aa were used. The length of F2A of 23aa was demonstrated to be the most efficient of the shorter versions of 2A, and deletion of just one amino acid from its N-terminus resulted in a substantial decrease in cleavage efficiency, whereas truncation to F2A_18_ essentially ablated cleavage: data apparently at variance with those previously reported for F2A_20_, where cleavage was efficient [10, 11; discussed below]. Cleavage efficiency was improved somewhat in constructs where the upstream gene was CherryFP (rather than GFP).

The exit tunnel of the ribosome accommodates 30–40 amino acids. For FMDV, the C-terminal region of protein 1D (immediately upstream of 2A) must have evolved to interact with the exit tunnel such that F2A (18aa long) mediates cleavage highly efficiently. In the case of artificial polyproteins, the ‘authentic’ upstream context of 2A is replaced with foreign gene or linker sequences which may create an unfavourable environment for ribosome ‘skipping’: their presence inside the exit tunnel may adversely affect the interaction of F2A with the ribosome. Low cleavage efficiency may arise, therefore, due to (i) the nature of the C-terminal region of the upstream gene, or, (ii) the manner in which it is linked to the shorter versions of F2A.

The data presented here is in agreement with many previously published studies that successfully used F2A and ‘2A-like’ sequences in a wide range of heterologous systems, notably, adoptive cell cancer gene therapies, genetic engineering of human stem cells and the co-expression of transcription factors for the induction of pluripotent stem cells [11,19,20,22-25,29-35]. Our findings also help explain previously published studies in which incomplete cleavage was observed. To avoid using longer 2A sequences (which remain attached at the C-terminus of the upstream protein), researchers used shorter versions of F2A. Conflicting cleavage efficiencies have been reported for 2As of the same lengths [11,14,19,22,25,27]. For example, Furler *et al*. [18] showed that within the context of a poliovirus vector, 2A-mediated cleavage was incomplete when F2A_24_ was used. Fang *et al*. [19] described similar results for the constitutive expression of full-length two-chain antibodies from a single ORF by linking the heavy and light antibody chains with F2A_24_. Different cleavage efficiencies depending on protein arrangement were observed when GFP, EGFP and hrGFP were co-expressed with cytochrome P450 2B1 (CYP2B1) via F2A_20 _[36]. In addition, Donnelly and co-workers, using F2A_20_ in GFP2AGUS and GUS2AGFP, also reported the presence of small amounts of uncleaved polyprotein [11]. With F2A_19_, cleavage was reported to be less efficient (70%) in [CFP-2A-PAC] context, with some uncleaved polyprotein still present [37], while de Felipe and co-workers [17] used an F2A_18_ version in the generation of retroviral vectors and also observed uncleaved polyprotein. The cleavage was, however, reported to be complete when the sequence was extended to 58aa [17]. Finally, Ma and Mitra used F2A_17_ to co-express GUS and CAT proteins in different arrangements and demonstrated that [GUS-2A-CAT] and [GUS-2A-GUS] were cleaved to completion, while [CAT-2A-GUS] was not [38].

Interestingly, we show here that pGFP-F2A_20_-CherryFP and pGFP-F2A_18_-CherryFP produced poor cleavage both *in vitro* and *in vivo*, apparently at variance with our own previously published data [10,11]. In these studies, the pGFP2AGUS construct contained the same upstream GFP gene and the same F2A_20_ sequence and produced efficient cleavage. Sequence comparison of pGFP-F2A_20_-CherryFP and pGFP2AGUS constructs shows that the differences are only observed in the sequence immediately upstream of 2A (RAKRSLE vs SGSRGAC, respectively). Ironically, this seven amino acid stretch is a part of neither F2A nor C terminus of the upstream GFP, but is actually composed of two different restriction sites introduced for cloning purposes during the construction of both plasmids. The effect of the differences in this particular region on the efficiency of 2A-mediated cleavage is the subject of a separate report [39].

Swapping the order of fluorescent ‘reporter’ proteins in our expression systems had no effect on the general pattern of cleavage efficiency. However, while two genes could be effectively co-expressed (irrespective of position) when linked by a longer version of F2A (30aa and longer), we did observe a small effect for the shorter F2A_20_ and F2A_18_ versions.

## Conclusions

In summary, F2As are used widely in biotechnology and biomedicine and we wished to identify the source(s) of apparent discrepancies in the literature with regards to cleavage efficiency. While constructs containing F2A of 58-23aa in length exhibited enhanced abilities for cleavage compared to the constructs with F2A of 22aa and shorter, with least efficient 2As being those of 20aa and 18aa, we suggest that researchers use F2A_30_ if opting for a shorter sequence. This 2A proved to be most optimal in terms of both length and cleavage efficiency, and, in our hands, was unaffected by the sequence of the upstream gene. A more extensive characterization of sequence requirements between the C-terminal region of the upstream protein and the N-terminal part of F2A is underway to test the speculation that this particularly interesting region of the sequence may have certain effect on 2A-mediated cleavage. Ultimately, the fine-tuning of the F2A sequence can help researchers in their efforts to efficiently co-express various multiple proteins for successful biomedical strategies.

## Methods

The following reagents were obtained from commercial suppliers: *Pfu* polymerase, T4 DNA ligase, restriction enzymes, JM109 competent cells, Quick *TnT* rabbit reticulocyte lysate kit (Promega, Madison, WI, USA); S^35^ methionine (MP Biomedicals, Santa Ana, CA, USA); EDTA-free protease inhibitor cocktail (Sigma-Aldrich Company Ltd, Dorset, UK); pcDNA3.1, anti-RFP, rabbit purified polyclonal antibody against Red Fluorescent Protein (1:1000 dilution), anti-GFP, mAb 3E6, anti-Green Fluorescent Protein, mouse monoclonal antibody (1:1000 dilution), anti-β-tubulin, mouse monoclonal antibody (1:2000 dilution), Lipofectamine2000 transfection reagent, iBlot Gel Transfer Stacks, nitrocellulose (Life Technologies Ltd, Paisley, UK); secondary HRP-conjugated polyclonal goat anti-mouse and anti-rabbit antibodies (Dako UK Ltd, Ely, UK); rabbit anti-FMDV 2A antiserum raised against the synthetic peptide NH2-LLNFDLLKLAGDVESNPGP-COOH; EZ-ECL Chemiluminescence Detection Kit for HRP (Geneflow Ltd, Elmhurst, UK); VECTASHIELD mounting medium with DAPI (VECTOR Laboratories Ltd, Peterborough, UK); Oligonucleotides were purchased from Integrated DNA Technologies (Coralville, Iowa, USA).

### Construction of plasmids

*pGFP-F2As-CherryFP constructs (F2As of 58, 50, 40, 30, 25–20, and 18aa,* sequences shown in Figure 1*)*. To make pGFP-F2A_58_-CherryFP construct, F2A_58_ sequence, obtained by digesting pSV7 plasmid (kindly provided by Sandra Varte) with NsiI and ApaI restriction enzymes, was cloned into pJC3 plasmid (pcDNA3.1-based with T2A linking GFP and CherryFP genes) in place of T2A sequence using these restriction sites. pGFP-F2A_58_-CherryFP construct was then used as a template to produce deletions of F2A. Forward oligonucleotides incorporating XhoI restriction site: (5′-CTCGAGAAGAGGGCCGAAACATACTGTCCAAGG-3′ for F2A_50_, 5′-CTCGAGTTGCTGGCAATCCACCCAACTGAAGCC-3′ for F2A_40_, 5′-GCGCTCGAGCACAAACAGAAAATTGTGGCACCGGTG-3′ for F2A_30_, 5′-GCGCTCGAGGTGGCACCGGTGAAACAGACTTTG-3′ for F2A_25_, 5′-GCGCTCGAGGCACCGGTGAAACAGACTTTG-3′ for F2A_24_, 5′-GCGCTCGAGCCGGTGAAACAGACTTTGAATTTTG-3′ for F2A_23_, 5′-GCGCTCGAGGTGAAACAGACTTTGAATTTTGAC-3′ for F2A_22_, 5′-GCGCTCGAGAAACAGACTTTGAATTTTGACCTTC-3′ for F2A_21_, 5′-GCGCTCGAGCAGACTTTGAATTTTGACCTTCTCAAG-3′ for F2A_20_, 5′-GCGCTCGAGTTGAATTTTGACCTTCTCAAGTTGGCG-3′ for F2A_18_) and a reverse oligonucleotide incorporating NotI restriction site (5′-TAGAAGGCACAGTCGAGGC-3′) were used to amplify F2As-CherryFP with 2As of various lengths. The PCR products were digested with XhoI and NotI restriction enzymes and cloned into pGFP-F2A_58_-CherryFP in place of F2A_58_-CherryFP sequence using XhoI and NotI restriction sites.

*pCherryFP-F2As-CherryFP constructs (F2As of 58, 50, 40, 30, 20, and 18aa).* Respective pGFP-F2As-CherryFP constructs were digested with BamHI and NsiI restriction enzymes to substitute GFP sequence with that of CherryFP. In order to do that, pJC3 plasmid was used as a template to amplify CherryFP gene using forward (5′-GCGGGATCCATGGTGAGCAAGGGCGAGGAGGATAAC -3′) and reverse (5′-GCGATGCATTTTGTACAATTCATCCATGCCGCCG -3′) primers incorporating, respectively, BamHI and NsiI restriction sites. PCR products were digested with BamHI and NsiI restriction enzymes and ligated into prepared vectors.

*pCherryFP-F2As-GFP constructs (F2As of 58, 50, 40, 30, 20 and 18aa).* Respective pCherryFP-F2As-CherryFP constructs were digested with ApaI and EcoRI restriction enzymes to substitute downstream CherryFP gene with that of GFP. pJC3 plasmid was used as a template to amplify GFP gene using forward (5′-GCGGGGCCCGATATCGTGTCCAAAGGGGAAGAG-3′) and reverse (5′-GCGGAATTCTTACTTATACAGCTCGTCCATGCCGAG-3′) primers incorporating ApaI and EcoRI restriction sites. PCR products were digested with ApaI and EcoRI restriction enzymes and ligated into prepared vectors.

### Transcription and translation

Plasmid constructs were used to programme Quick *TnT* Coupled Transcription/ Translation reticulocyte lysate systems (Promega) and the reactions repeated three times. Translation reactions (10 μl) were performed according to the manufacturer’s instructions. Briefly, 20 ng of plasmid DNA was used to programme rabbit reticulocyte lysates containing ^35^S methionine (10 μCi) and the reactions were incubated at 30°C for 1 h. Translation reactions were stopped by the addition of 2x protein dissociation buffer. Protein products were analysed by 12% SDS-PAGE. Controls of TnT reactions programmed with ‘empty’ vectors produced no translation products (data not shown).

### Expression in HeLa cells

HeLa cells were maintained in DMEM medium supplemented with 10% FCS. For western blotting, the cells were transfected in 60 mm dishes (pre-plated 20 h earlier to 60% confluency) with 1.5 μg of plasmid DNA and 7 μl of Lipofectamine2000 (Invitrogen) in a final volume of 400 μl OptiMEM. Transfection mix was added to 4 ml of antibiotic-free serum-containing medium and cells were incubated for a total of 30 h post-transfection. All transfections were performed in triplicate. Controls with ‘empty’ expression vector transfected cells produced no translation products (western blotting) or fluorescence (data not shown).

### Western blotting

30 h post-transfection, cells were washed twice with 1 ml of PBS and harvested in 1 ml of PBS by centrifugation at 2000 rpm for 5 min. Whole-cell lysates were prepared in 70 μl of radioimmuno precipitation assay buffer, RIPA (150 mM NaCl, 10 mM Tris pH 7.4, 1% Triton X-100, 1% Na deoxycholate, 0.1% SDS, and freshly added 1/20 volume of EDTA-free protease inhibitor cocktail) by incubation on ice for 30 min and centrifugation at 12000 rpm for 20 min at 4°C. Cellular debris was discarded and supernatants containing proteins were analysed by 12% SDS-PAGE. The proteins were then transferred to a nitrocellulose membrane, which was probed with anti-GFP, anti-CherryFP or anti-FMDV 2A primary antibodies overnight after blocking in PBST containing 5% milk for 1 h. Following overnight incubation, the membranes were washed three times in PBST. Detection of bound primary antibodies was performed with respective HRP-secondary antibodies (Dako) in PBST containing 5% milk for 1 h, the membranes were washed three times with PBST, rinsed in deionised water and subjected to enhanced chemiluminescence by incubating it in freshly-prepared visualisation solution for 2 min. Membrane was exposed to an autoradiograph film for 3 to 45 seconds.

### Fluorescence microscopy

Transfection of HeLa cells was performed as described above for western blotting, however, 0.5 μg of plasmid DNA and 5 μl of Lipofectamine2000 in a final volume of 200 μl OptiMEM were added to 2 ml of media in each well of a 6-well plate containing coverslips. 30 h post-transfection the cells were washed twice with 1 ml of PBS, fixed with 1 ml of ice-cold 100% methanol for 10 min and washed again with 1 ml of PBS. The coverslips were mounted in VECTASHIELD (an antifade mounting medium, VECTOR Laboratories). The images were acquired with Deltavision microscope using 100 × objective using Resolve 3D software. Experiments were done in triplicates.

## Competing interests

The authors declared that they have no competing interests.

## Authors’ contributions

EM carried out cloning, sequence alignment*, in vitro TnT* analysis, cell transfections, Western blotting, fluorescence analysis, and drafted the manuscript. JN participated in cloning and *in vitro TnT* analysis. MDR helped to draft the manuscript. All authors read and approved the final manuscript.
